# Association between Eight Functional Polymorphisms and Haplotypes in the Cholesterol Ester Transfer Protein (CETP) Gene and Dyslipidemia in National Minority Adults in the Far West Region of China

**DOI:** 10.3390/ijerph121215036

**Published:** 2015-12-16

**Authors:** Shuxia Guo, Yunhua Hu, Yusong Ding, Jiaming Liu, Mei Zhang, Rulin Ma, Heng Guo, Kui Wang, Jia He, Yizhong Yan, Dongsheng Rui, Feng Sun, Lati Mu, Qiang Niu, Jingyu Zhang, Shugang Li

**Affiliations:** Department of Public Health and Key Laboratory of Xinjiang Endemic and Ethnic Diseases of the Ministry of Education, Shihezi University School of Medicine, Shihezi 832002, China; hyh6133@sina.com (Y.H.); 13399931625@163.com (Y.D.); liujiaming@shzu.edu.cn (J.L.); zmberry@foxmail.com (M.Z.); marulin@126.com (R.M.); guoheng@shzu.edu.cn (H.G.); kwang311@hotmail.com (K.W.); hejia123.shihezi@163.com (J.H.); erniu19880215@sina.com (Y.Y.); ruidongsheng@gmail.com (D.R.); sunfeng@bjmu.edu.cn (F.S.); murat08123@163.com (L.M.); niuqiang1214@163.com (Q.N.); yfyxxzjy@126.com (J.Z.); lishugang@ymail.com (S.L.)

**Keywords:** CETP gene, polymorphism, haplotype, dyslipidemia, national minority, SNPs

## Abstract

We have investigated the relationship between the polymorphisms and haplotypes in the CETP gene, and dyslipidemia among the Xinjiang Kazak and Uyghur populations in China. A total of 712 patients with dyslipidemia and 764 control subjects of CETP gene polymorphism at rs12149545, rs3764261, rs1800775, rs711752, rs708272, rs289714, rs5882, and rs1801706 loci were studied by the Snapshot method, linkage disequilibrium analysis and haplotype construction. The results are as follows: (1) the minor allele of eight loci of frequencies in the two groups were different from other results of similar studies in other countries; (2) In the linear regression analysis, the HDL-C levels of rs708272 TT, rs1800775 AA, rs289714 CC and rs711752 AA genotypes were significantly higher than those of other genotypes, however, the rs3764261 GG and rs12149545 GG genotypes were significantly lower than those of other genotypes in the two ethnic groups. The HDL-C levels of the rs12149545 GG genotype were lower than those of other genotypes; (3) in the control group, the rs708272 CT genotype of TG levels were lower than in the CC genotype, the T genotype of LDL-C levels were lower than in the CC genotype, and the HDL-C levels were higher than in the CT genotype; the rs1800775 AC genotype of TG levels were higher than in the AA genotype, the rs711752 AG genotype of TG levels were lower than in the GG genotype, the AA genotype LDL-C levels were lower than in the GG genotype, and the HDL-C levels were higher than in the AG genotype; the rs1800775 AC genotype of TG levels were higher than in the AA genotype. In the dyslipidemia group, the rs708272 TT genotype of TC and LDL-C levels were higher than in the CT genotype and the rs3764261 TT genotype of TC levels were higher than in the GG genotype. The rs711752 AA genotype of TC and LDL-C levels were higher than in the AG genotype, and the rs12149545 AA genotype of TC and LDL-C levels were higher than in the GG genotype; (4) perfect Linkage Disequilibrium was observed for two sets of two SNPs: rs3764261 and rs12149545; rs711752 and rs708272. (5) Using SHEsis software analysis, the five A/T/A/A/T/C/A/G, A/T/A/A/T/T/G/A, G/G/A/G/C/C/G/G, G/G/C/G/C/C/A/G and G/G/C/G/C/T/G/G haplotypes were between dyslipidemia group and control group statistically significantly different (*p* < 0.05 in each case). The polymorphism of CETP genes rs708272, rs3764261, rs1800775, rs711752, rs12149545 was closely related to the dyslipidemia in the Xinjiang Uyghur and Kazakh ethnic groups; and the rs708272 T, rs3764261 T, rs711752 A, and rs12149545 A alleles could reduce risk of dyslipidemia in the Uyghur and Kazakh populations, however, the rs1800775 C allele showed risk factors.

## 1. Introduction

At present, dyslipidemia is a common health problem around the world [1], including in China [2]. The majority of studies have demonstrated that lipoprotein concentrations and plasma lipids are important risk factors for atherosclerosis and related vascular diseases, which are the main causes of death in China and other regions of the world [3]. Dyslipidemia is characterized by an accumulation of lipoprotein abnormalities, including low high-density lipoprotein cholesterol (HDL-C), high serum triglycerides (TG), high total cholesterol (TC), and increased small low-density lipoprotein cholesterol (LDL-C). The prevalence of dyslipidemia has increased not only in China, but also worldwide. It was reported that in 2008 the dyslipidemia prevalence among Swiss Caucasians was 34.2% in Lausanne [4], and that in 2010 the high TC prevalence and low HDL-C prevalence were 23% and 47% respectively in the South Asian ethnicity in India [5]. An American study [6] showed that, from 1999 to 2006 the high TG prevalence in adults had increased from 24.7% to 27.6%. Further, it was found that in 2011 the dyslipidemia prevalence was over 55% and without racial differences in USA [7].

China is a multi-ethnic country, and in the Xinjiang Uyghur Autonomous Region alone there are 47 ethnic groups, among which Uyghur and Kazakh are the first and second largest populations, respectively. We previously showed that the prevalence of dyslipidemia among the Uyghur and Kazakh populations in Xinjiang were 42.4% and 31.8%, respectively [8], higher than the 28.2% of the local Han population, and significantly higher than 18.6%, the reported one for average adults in China. These figures indicated an unusually high prevalence of dyslipidemia in the Uyghur and Kazakh populations.

Dyslipidemia is a common result of genetic and metabolic factors caused by social life disorders. Epidemiological studies have reported that about 50% of the variation in HDL-C, LDL-C, and TC levels is genetically determined [9]. Since 2008, genome-wide association (GWA) studies of plasma lipid levels have further identified several common variants associated with plasma lipid levels [10,11,12]. A number of newly identified genes are potential new drug targets. Therefore, these recent genetic advances have broadened our understanding of the basic metabolic pathways and may improve patient classification, disease diagnosis and treatment strategies [11]. In fact, the most remarkable studies of gene synthesis, metabolic pathways and its variants of dyslipidemia have been consistently associated with HDL-C. The cholesterol ester transfer protein (CETP) is a key plasma protein that mediates the transfer of esterified cholesterol from HDL to ApoB containing particles in exchange for TG [13]. It is found that CETP gene located on chromosome 16q21 is highly polymorphic [14]. However, due to the known differences in genome-wide linkage disequilibrium patterns between different ethnic groups, it's still unclear whether the loci identified in European GWA studies also exert similar effects on lipid levels among the Uyghur and Kazakh in Western China. Accordingly, we investigated eight (rs12149545, rs3764261, rs1800775, rs711752, rs708272, rs289714, rs5882, rs1801706) single nucleotide polymorphisms (SNPs) in the CETP gene in a sample consisting of 1476 individuals (dyslipidemia: 712 and normal control: 764) from the Xinjiang ethnic minorities to determine the association between genetic variations in the CETP gene and dyslipidemia in this national minority population.

As the first study of the association between the CETP multiple loci and dyslipidemia in Western China, we investigated the possible pathogenesis of dyslipidemia from a genetic perspective. The findings may provide a new way of prognosis, prevention and treatment of dyslipidemia at the genetic level in the Uyghur and Kazakh ethnic groups, as well a genetic basis for the prevention and treatment of cardiovascular and cerebrovascular diseases.

## 2. Materials and Methods

The Institutional Ethics Review Board at the First Affiliated Hospital of Shihezi University School of Medicine approved this study (IERB No. SHZ2010LL01). Standard university hospital guidelines including informed consent, confidentiality, voluntary participation, and anonymity were followed. All participants gave written informed consent before the study began.

### 2.1. Study Population

This study was conducted from 2009 to 2010. Cases (3049) aged 18 years and above among Uyghur nationality residents of Jiashi County and 5692 cases aged 18 years and above among Kazakh nationality residents in Yili region Xinyuan County of Xinjiang Kashgar Prefecture were selected using the stratifying cluster sampling method. Xinyuan County is ~3692 km (2294 miles) and Jiashi County is ~4329 km (2690 miles) away from Beijing. All the research object had lived >15 years in the region. Various biochemical parameters were tested for all blood samples according to the 2007 China Adult Dyslipidemia Prevention Guide [15] of dyslipidemia. Seven hundred and twelve patients with dyslipidemia were randomly assigned to the case group and a total of 764 cases with normal blood lipids were randomly assigned to the control group using the group matching sex, age and ethnic method, Subjects with hypertension, diabetes or obesity were excluded from the study.

### 2.2. Basic Data Collection and Dyslipidemia Diagnosis

All study subjects completed a demographic information survey questionnaire, including details of disease(s), family, smoking and drinking habits, diet, and physical exercise. The subjects’ sitting blood pressure, height, weight, waist circumference (WC) and hip circumference (HC) were also measured and recorded. Laboratory analyses of blood samples included tests for fasting TG, LDL-C, TC, HDL-C, and fasting plasma glucose (FPG), etc., all of which were analyzed using an automatic biochemical analyzer (AU400, Olympus: Tokyo, Japan). Based on the China Adult Dyslipidemia Prevention Guide (2007) [15] dyslipidemia was defined as presentation of any one or more of the following four items: (1) TG ≥ 2.26 mmol/L (200 mg/dL) (2) HDL-C < 1.04 mmol/L (40 mg/dL) (3) LDL-C ≥ 4.14 mmol/L (160 mg/dL) (4) TC ≥ 6.22 mmol/L (240 mg/dL).

### 2.3. DNA Extraction

Fasting venous blood (200 µL) was taken from each study subject and a non-centrifugal columnar blood genomic DNA isolation kit (Tiangen, Beijing, China) was used to extract the whole blood genomic DNA. Extracted DNA was verified by gel electrophoresis (0.7% agarose). A NanoDrop spectrophotometer (NanoDrop technologies, Inc.: Wilmington, DE, USA) was used for quantitative determination of DNA concentration and purity: concentration ≥ 30 ng/µL and purity levels (OD260/OD280) of 1.7–2.0 were considered acceptable. Samples that met these criteria were diluted to 10–30 ng/µL using double-distilled water and stored at −80 °C until use.

### 2.4. PCR Amplification

Primers were designed using the Mysequenom tool (www.mysequenom.com/Home) and AssayDesigner3.0 software (SEQUENOM, Inc.: San Diego, CA, USA). Final PCR reaction volumes were 15 µL, which included 1 µL DNA samples, 0.3 µL dNTPs, 7.4 µL water, 1.5 μL 10 × PCR buffer, 1.5 µL MgCl_2_, 0.3 µL Taq enzymes, and 3 μL PCR amplification primer mixture. Cycling conditions were as follows: predegeneration at 94 °C for 4 min; followed by 35 cycles of denaturation at 94 °C for 20 s, annealing at 56 °C for 30 s, and extension at 72 °C for 1 min. A final extension step was carried out at 72 °C for 3 min, after which samples were maintained at 4 °C. Reactions were set up in an ice bath and each PCR experiment included a negative control reaction.

### 2.5. PCR Products Purification

Shrimp alkaline phosphatase (SAP) was used to remove excess dNTPs from samples after PCR. This step served to ensure the accuracy of single-base extension. The final SAP reaction volumes were 5.0 mL, which included 0.5 µL 10 × SAP buffer, 2 μL PCR product, 2 µL double-distilled water, and 0.5 µL SAP enzyme. Reactions were carried out by incubation at 37 °C for 40 min, followed by incubation at 85 °C for 5 min. The reaction products were stored at 4 °C.

### 2.6. Single-Base Extension

For single-base extension reactions, final reaction volumes were 6.0 µL, which included 0.5 μL Snapshot reagent, 2.5 µL water, 1 µL primer mix, 2 uL purified PCR products. Reaction conditions were as follows: denaturation at 94 °C for 30 s; followed by 40 cycles of 94 °C for 5 s, 52 °C for 5 s, and finally 52 °C for 5 s. Reaction products were stored at 4 °C.

### 2.7. Genotyping Analysis

Take 1 µL reaction product plus 9 µL HIDI, 95 °C denaturation 3 min, immediately ice-water bath, all representative SNP genotyping experiments were done using TaqMan technology on an ABI3730XL system (Applied Biosystems: Carlsbad, CA, USA). T gene-mapper was used to complete the classification and output the results.

### 2.8. Statistical Analysis

We adopted the Epidata 3.1 software to manage the study data and the double entry method was used for data entry and logical error detection. Hardy-Weinberg balance was calculated to confirm the group representation of the samples and SPSS statistical software version 20.0 for Windows (IBM: Almon, NY, USA) was used for all data analysis. Descriptive statistics were calculated to characterize the participants and study variables. The gene counting method was used to calculate the genotype and allele frequencies, chi-square testing was used for comparisons between groups, and t-testing was used to assess clinical variables between groups. Linear regression analysis of linear relationship between variables; Logistic regression analysis was used to assess risk factors for which odds ratios (ORs) and 95% CI were calculated. Significance was set at 0.05. SHEsis software was used to perform the Hardy-Weinberg equilibrium test, haplotype construction and statistical analysis (the haplotype were deleted for the two groups of frequencies were less than 0.01) [16].

## 3. Results

### 3.1. Assessment of General Data and Clinical Biochemical Indices

A total of 764 (male: 375; female: 389) normal control and 712 (male: 354; female: 358) dyslipidemia subjects were included in the present study. The mean ages in the normal control and dyslipidemia groups were 43.60 ± 15.32 and 45.16 ± 15.08 years, respectively. The general clinical data for the subjects in each group are shown in Table 1. There were no significant differences in the gender, age, LPa, smoking, and drinking habits of each group (*p* > 0.05). In the dyslipidemia group, the mean height, weight, BMI, WC, HC, WHR, SBP, DBP, TG, TC, LDL-C, FPG, ApoB, ApoB/ApoA, and family history were higher than in the normal control groups while mean HDL-C, ApoA was lower in the dyslipidemia group than in the normal control group (*p* < 0.05 in each case).

**Table 1:** Comparison of clinical and biochemical indicators across the normal control and dyslipidemia groups.

Clinical and Biochemical Indices	Group		χ^2^/t	*p* Value
	Normal Control	Dyslipidemia		
*n* (Male/Female)	764 (375/389)	712 (354/358)	0.060	0.807
Age (year)	43.60 ± 15.32	45.16 ± 15.08	−1.955	0.051
Height (cm)	161.04 ± 8.78	162.17 ± 9.06	−2.408	0.016 *
Weight (kg)	58.68 ± 10.99	67.05 ± 13.87	−12.813	<0.001 *
BMI (kg/m^2^)	22.55 ± 3.48	25.38 ± 4.20	−14.040	<0.001 *
WC (cm)	81.23 ± 9.97	89.99 ± 11.90	−15.264	<0.001 *
HC (cm)	93.84 ± 7.60	98.62 ± 8.49	−11.330	<0.001 *
WHR	0.87 ± 0.07	0.91 ± 0.08	−12.068	<0.001 *
SBP (mmHg)	128.68 ± 23.11	134.11 ± 24.08	−4.399	<0.001 *
DBP (mmHg)	80.87 ± 13.76	84.30 ± 14.55	−4.634	<0.001 *
TG (mmol/L)	0.80 ± 0.28	2.16 ± 1.74	−21.397	<0.001 *
TC (mmol/L)	4.00 ± 0.71	4.90 ± 1.58	−14.261	<0.001 *
LDL-C (mmol/L)	2.02 ± 0.52	2.73 ± 1.07	−16.448	<0.001 *
HDL-C (mmol/L)	1.45 ± 0.29	1.09 ± 0.43	19.517	<0.001 *
FPG (mmol/L)	4.54 ± 1.28	4.80 ± 1.73	−3.300	0.001 *
LPa (mg/L)	259.51 ± 321.85	251.37 ± 348.31	0.439	0.661
ApoA (g/L)	1.38 ± 0.23	1.23 ± 0.37	9.104	<0.001 *
ApoB (g/L)	0.73 ± 0.23	1.02 ± 0.41	−16.500	<0.001 *
ApoB/ApoA	0.53 ± 0.17	0.85 ± 0.52	−15.068	<0.001 *
Smoking (Yes/No)	(205/557)	(215/489)	2.368	0.124
Drinking (Yes/No)	(70/693)	(64/639)	0.002	0.963
Family history (Yes/No)	(219/545)	(249/463)	6.770	0.009 *

Abbreviations: BMI, body mass index; WC, waist circumference; HC, hip circumference; WHR, waist-to-hip ratio; SBP, systolic blood pressure; DBP, diastolic blood pressure; TG, triglyceride; TC, total cholesterol; LDL-C, low-density lipoprotein-cholesterol; HDL-C, high-density lipoprotein-cholesterol; FPG, fasting plasma glucose; LPa, lipoprotein(a); ApoA, apolipoprotein A; ApoB, apolipoprotein B; dyslipidemia group *vs.* normal control group, * *p* < 0.05; Data represent mean ± SD.

### 3.2. Hardy-Weinberg Equilibrium Testing of and Success Rate of Gene Frequencies

In our study, eight SNPs in the CETP gene were genotyped, and all loci were in agreement with the Hardy-Weinberg equilibrium (all *p* > 0.05), indicating that eight loci of the CETP gene reached genetic equilibrium and the samples were thus indeed representative of the Uyghur and Kazakh population. For the eight SNPs, the success rates were 99.2% (rs5882), 99.2% (rs289714), 99.3% (rs708272), 99.3% (rs711752), 99.0% (rs1800775), 99.4% (rs1801706), 99.3% (rs3764261), 99.3% (rs121459545). Analysis of the missing data showed no significant differences between dyslipidemia cases and normal controls.

### 3.3. Association of CETP Snps in Dyslipidemia and Normal Control Individuals in Xinjiang National Minorities

The CETP gene eight SNPs allele and genotype distributions of the variants in dyslipidemia and normal control groups are summarized in Table 2. Based on the adjustment of age, sex and body mass index, the risk degree was evaluated by dyslipidemia (0 = no, 1 = yes) as the dependent variable, and the main allele was the control, and the CETP gene was analyzed by single factor logistic regression analysis, OR and 95%CI. There were five loci allelic showed associations with dyslipidemia as follows: rs708272 (*p* = 0.047): of these SNPs, carriers of the T allele of rs708272 had significant lower risk of dyslipidemia (OR = 0.867, 95% CI = 0.753–0.998) compared with carriers of the major C allele; rs711752 (*p* = 0.045), carriers of the A allel of rs711752 had significant lower risk of dyslipidemia (OR = 0.861, 95% CI = 0.745–0.996) compared with carriers of the major G allele; rs1800775 (*p* = 0.039), the people carriers of the C allele of rs1800775 had significant higher risk of dyslipidemia (OR = 1.161, 95% CI = 1.007–1.339) compared with carriers of the major A allele; rs3764261 (*p* = 0.001): carriers of the T alleld of rs3764261 had significant lower risk of dyslipidemia (OR = 0.754, 95% CI = 0.642–0.886) compared with carriers of the major G allelde; rs12149545 (*p* = 0.0003), carriers of the A allele of rs12149545 had significant lower risk of dyslipidemia (OR = 0.744, 95% CI = 0.633–0.876) compared with carriers of the major G allele. The remaining bits in the distribution of genotypes point difference between the two groups were not statistically significant.

**Table 2:** Association of CETP SNPs in Dyslipidemia and Normal Control individuals in Xinjiang national minority.

SNP	Chr	Gene	M/m	Group(s)	Genotype Distribution *n* (%)						Allelic Distribution *n* (%)				χ^2^	*p* ^b^	OR	95% CI for OR
					R/R ^a^		C/R ^a^		C/C ^a^		R ^a^		C ^a^					
rs5882	16	CETP	A/G	Dyslipidemia	99	(14.0)	310	(43.8)	299	(42.2)	508	(35.9)	908	(64.1)				
				Normal Control	111	(14.7)	340	(44.9)	306	(40.4)	562	(37.1)	952	(62.9)	0.469	0.494	0.950	0.820–1.101
rs289714	16	CETP	T/C	Dyslipidemia	46	(6.5)	275	(38.8)	387	(54.7)	367	(25.9)	1049	(74.1)				
				Normal Control	35	(4.6)	286	(37.8)	436	(57.6)	356	(23.5)	1158	(76.5)	2.343	0.126	1.143	0.963–1.356
rs708272	16	CETP	C/T	Dyslipidemia	156	(22.0)	322	(45.4)	231	(32.6)	634	(44.7)	784	(55.3)				
				Normal Control	186	(24.6)	362	(47.8)	209	(27.6)	734	(48.5)	780	(51.5)	3.928	0.047	0.867	0.753–0.998
rs711752	16	CETP	G/A	Dyslipidemia	156	(22.0)	323	(45.6)	230	(32.4)	635	(44.8)	783	(55.2)				
				Normal Control	186	(24.6)	362	(47.8)	209	(27.6)	734	(48.5)	780	(51.5)	3.787	0.045	0.861	0.745–0.996
rs1800775	16	CETP	A/C	Dyslipidemia	152	(21.5)	318	(45.0)	236	(33.4)	622	(44.1)	790	(55.9)				
				Normal Control	132	(17.5)	343	(45.4)	281	(37.2)	607	(40.1)	905	(59.9)	4.255	0.039	1.161	1.007–1.339
rs1801706	16	CETP	G/A	Dyslipidemia	5	(0.7)	133	(18.7)	572	(80.6)	143	(10.1)	1277	(89.9)				
				Normal Control	9	(1.2)	151	(19.9)	597	(78.9)	169	(11.2)	1345	(88.8)	0.752	0.386	0.900	0.709–1.142
rs3764261	16	CETP	G/T	Dyslipidemia	51	(7.2)	264	(37.2)	394	(55.6)	366	(25.8)	1052	(74.2)				
				Normal Control	72	(9.5)	334	(44.1)	351	(46.4)	478	(31.6)	1036	(68.4)	11.718	0.001	0.754	0.642–0.886
rs12149545	16	CETP	G/A	Dyslipidemia	50	(7.1)	251	(35.4)	408	(57.5)	351	(24.8)	1067	(75.2)				
				Normal Control	68	(9.0)	329	(43.5)	360	(47.6)	465	(30.7)	1049	(69.3)	12.665	0.0003	0.744	0.633–0.876

Abbreviations: Chr = chromosome; M indicates major allele; m indicates minor allele. ^a^, R/R, homozygous for minor allele; C/R, heterozygous for minor allele; C/C, homozygous for common allele. ^b^, *p* values were adjusted for age, sex and BMI.

### 3.4. SNPs Associated with TG, TC, LDL-C and HDL-C

The results showed that there are five loci SNPs associated with TG, TC, LDL and HDL-C that show significant differences. In control group, the rs708272 CT genotype TG levels were lower than in the CC genotype, TT genotype LDL-C levels were lower than in the CC genotype, and the HDL-C levels were higher than in the CT genotype; the rs1800775 AC genotype TG levels were higher than in the AA genotype. The rs711752 AG genotype TG levels were lower than in the GG genotype, AA genotype LDL-C levels were lower than in the GG genotype, and the HDL-C levels were higher than in the AG genotype; the rs1800775 AC genotype TG levels were higher than in the AA genotype. In the dyslipidemia group, the rs708272 TT genotype TC and LDL-C levels were higher than in the CT genotype, and the rs3764261 TT genotype TC levels were higher than in the GG genotype. The rs711752 AA genotype TC and LDL-C levels were higher than in the AG genotype, and the rs12149545 AA genotype TC and LDL-C levels were higher than in the GG genotype (*p* < 0.05 in each case). The remaining loci in the distribution of genotypes and TG, TC, LDL-C and HDL-C in the two groups were not statistically significant (Table 3).

**Table 3:** Association of the Polymorphisms in CETP gene with TG, TC, LDL-C and HDL-C in two groups.

SNP	Group	Genotypes	*n*	TG	TC	LDL-C	HDL-C
rs708272	Normal Control	CC	209	0.83 ± 0.31 *	4.04 ± 0.77	2.08 ± 0.55 *	1.45 ± 0.31
		CT	362	0.77 ± 0.27 *	3.99 ± 0.65	2.00 ± 0.51	1.43 ± 0.28 *
		TT	186	0.80 ± 0.28	3.96 ± 0.74	1.96 ± 0.52 *	1.51 ± 0.30 *
	Dyslipidemia	CC	231	2.24 ± 2.42	4.89 ± 1.63	2.74 ± 1.09	1.07 ± 0.46
		CT	322	2.07 ± 1.15	4.78 ± 1.54 *	2.65 ± 1.02 *	1.07 ± 0.40
		TT	156	2.25 ± 1.57	5.15 ± 1.57 *	2.88 ± 1.12 *	1.14 ± 0.43
rs1800775	Normal Control	AA	281	0.83 ± 0.27 *	4.01 ± 0.65	1.99 ± 0.52	1.48 ± 0.30
		AC	343	0.77 ± 0.28 *	3.99 ± 0.67	2.02 ± 0.52	1.44 ± 0.30
		CC	132	0.80 ± 0.31	3.96 ± 0.90	2.04 ± 0.56	1.45 ± 0.26
	Dyslipidemia	AA	236	2.15 ± 1.42	5.02 ± 1.59	2.80 ± 1.09	1.12 ± 0.42
		AC	318	2.22 ± 1.95	4.88 ± 1.57	2.73 ± 1.08	1.06 ± 0.38
		CC	152	2.08 ± 1.77	4.77 ± 1.57	2.63 ± 1.01	1.08 ± 0.52
rs3764261	Normal Control	GG	351	0.81 ± 0.29	4.00 ± 0.73	2.05 ± 0.53	1.44 ± 0.29
		GT	334	0.78 ± 0.27	3.99 ± 0.67	1.98 ± 0.53	1.47 ± 0.31
		TT	72	0.83 ± 0.30	4.03 ± 0.76	2.02 ± 0.49	1.46 ± 0.24
	Dyslipidemia	GG	394	2.15 ± 1.97	4.82 ± 1.59 *	2.68 ± 1.05	1.08 ± 0.44
		GT	264	2.22 ± 1.47	4.93 ± 1.59	2.75 ± 1.04	1.10 ± 0.42
		TT	51	2.04 ± 1.08	5.31 ± 1.41 *	2.99 ± 1.26	1.10 ± 0.35
rs711752	Normal Control	GG	209	0.83 ± 0.31 *	4.04 ± 0.77	2.08 ± 0.55 *	1.45 ± 0.31
		AG	362	0.77 ± 0.27 *	3.99 ± 0.65	2.00 ± 0.51	1.43 ± 0.28 *
		AA	186	0.80 ± 0.28	3.96 ± 0.74	1.96 ± 0.52 *	1.51 ± 0.30 *
	Dyslipidemia	GG	230	2.24 ± 2.42	4.89 ± 1.63	2.74 ± 1.09	1.07 ± 0.46
		AG	323	2.07 ± 1.15	4.78 ± 1.54 *	2.65 ± 1.02 *	1.07 ± 0.40
		AA	156	2.25 ± 1.57	5.15 ± 1.57 *	2.88 ± 1.12 *	1.14 ± 0.43
rs12149545	Normal Control	GG	360	0.81 ± 0.29	4.00 ± 0.73	2.05 ± 0.53	1.44 ± 0.29
		AG	329	0.78 ± 0.27	3.99 ± 0.67	1.98 ± 0.53	1.47 ± 0.31
		AA	68	0.83 ± 0.30	4.02 ± 0.77	2.01 ± 0.48	1.46 ± 0.24
	Dyslipidemia	GG	408	2.18 ± 1.96	4.84 ± 1.57 *	2.69 ± 1.05 *	1.07 ± 0.44
		AG	251	2.17 ± 1.47	4.91 ± 1.62	2.74 ± 1.05	1.10 ± 0.42
		AA	50	2.05 ± 1.09	5.33 ± 1.41 *	3.01 ± 1.26 *	1.10 ± 0.36

*, *p* < 0.05.

### 3.5. Linkage Disequilibrium (LD) Tests

Pairwise LD between the eight tagSNPs is shown in Table 4. Eight SNPs in the CETP gene (rs12149545–rs3764261–rs1800775–rs711752–rs708272–rs289714–rs5882–rs1801706) were in linkage disequilibrium with D' ranging from 0.04 to 1.00 and r2 ranging from 0.00 to 0.99. Perfect LD observed for two sets of two SNPs was for rs3764261 and rs12149545; rs711752 and rs708272.

We detected associations with haplotypes to a similar or even greater degree than with SNPs. A closer analysis revealed that of the 1476 possible 8-polymorphism haplotypes (rs12149545/rs3764261/rs1800775/rs711752/rs708272/rs289714/rs5882/rs1801706), 46 were estimated to be present. These eight polymorphisms generated seventeen main haplotypes in the dyslipidemia and normal control groups, respectively, accounting for 95.3% and 95.2% of all the haplotypes observed (Table 5).

**Table 4:** Linkage disequilibrium test of CETP gene.

	rs12149545	rs3764261	rs1800775	rs711752	rs708272	rs289714	rs5882	rs1801706
**rs12149545**	—	1.000	0.930	0.977	0.975	0.744	0.116	0.376
**rs3764261**	0.954	—	0.928	0.913	0.910	0.677	0.124	0.386
**rs1800775**	0.241	0.252	—	0.919	0.919	0.214	0.637	0.377
**rs711752**	0.421	0.385	0.537	—	1.000	0.229	0.461	0.417
**rs708272**	0.419	0.383	0.536	0.999	—	0.230	0.462	0.418
**rs289714**	0.070	0.061	0.021	0.015	0.015	—	0.043	0.908
**rs5882**	0.009	0.011	0.169	0.139	0.140	0.000	—	0.972
**rs1801706**	0.006	0.007	0.012	0.024	0.024	0.032	0.195	—

Note: the upper triangle is D′ value and the lower triangle is *r*^2^ value.

**Table 5:** The distribution of haplotypes of eight SNPs in CETP gene between the cases and controls in Xinjiang Minority nationality.

Haplotype	Dyslipidemia (%)	Normal Control (%)	χ^2^	Fisher’s *p*	Pearson’s *p*	OR (95% CI)
A/T/A/A/T/C/A/G	9.27 (0.007)	24.01 (0.016)	5.679	0.017	0.017	0.408 (0.190–0.874)
A/T/A/A/T/T/A/G	185.76 (0.132)	231.57 (0.154)	2.911	0.087	0.087	0.834 (0.676–1.028)
A/T/A/A/T/T/G/A	12.67 (0.009)	30.16 (0.020)	6.144	0.013	0.013	0.443 (0.229–0.858)
A/T/A/A/T/T/G/G	123.60 (0.088)	143.97 (0.096)	0.556	0.456	0.455	0.908 (0.705–1.170)
G/G/A/A/T/C/G/G	103.20 (0.073)	91.84 (0.061)	1.741	0.187	0.186	1.217 (0.909–1.628)
G/G/A/A/T/T/A/G	67.86 (0.048)	51.83 (0.034)	3.480	0.062	0.062	1.419 (0.981–2.054)
G/G/A/A/T/T/G/A	65.20 (0.046)	79.17 (0.053)	0.623	0.430	0.430	0.873 (0.624–1.223)
G/G/A/A/T/T/G/G	25.71 (0.018)	30.36 (0.020)	0.144	0.704	0.704	0.903 (0.531–1.534)
G/G/A/G/C/C/A/G	12.52 (0.009)	16.42 (0.011)	0.303	0.581	0.581	0.813 (0.388–1.702)
G/G/A/G/C/C/G/G	5.36 (0.004)	15.33 (0.010)	4.206	0.040	0.040	0.371 (0.138–0.995)
G/G/A/G/C/T/A/G	67.63 (0.048)	79.30 (0.053)	0.338	0.561	0.561	0.906 (0.649–1.264)
G/G/A/G/C/T/G/A	18.04 (0.013)	9.62 (0.006)	3.182	0.074	0.074	2.017 (0.919–4.426)
G/G/A/G/C/T/G/G	50.09 (0.036)	59.51 (0.040)	0.323	0.569	0.569	0.895 (0.610–1.313)
G/G/C/G/C/C/A/G	196.39 (0.139)	167.58 (0.111)	5.255	0.021	0.021	1.294 (1.038–1.615)
G/G/C/G/C/T/A/G	329.51 (0.234)	352.52 (0.234)	0.001	0.978	0.977	0.998 (0.839–1.186)
G/G/C/G/C/T/G/A	27.00 (0.019)	32.64 (0.022)	0.233	0.629	0.629	0.881 (0.526–1.475)
G/G/C/G/C/T/G/G	41.67 (0.030)	16.67 (0.011)	12.697	0.0003	0.0003	2.723 (1.535–4.829)

The 29 remaining haplotypes were inferred from the data at a frequency of less than 0.01. The A/T/A/A/T/T/A/G, G/G/C/G/C/C/A/G and G/G/C/G/C/T/A/G haplotypes were most prevalent with estimated relative frequencies respectively dyslipidemia (0.132, 0.139, 0.234) and normal control group (0.154, 0.111, 0.234).

Simultaneously, we investigated five kinds of the CETP haplotypes that were significantly associated with risk of dyslipidemia and normal control groups (A/T/A/A/T/C/A/G, *p* = 0.017, OR = 0.408, confidence interval (95% CI) = 0.190–0.874; A/T/A/A/T/T/G/A, *p* = 0.013, OR = 0.443, 95% CI = 0.229–0.858; G/G/A/G/C/C/G/G, *p* = 0.04, OR = 0.371, 95% CI = 0.138–0.995; G/G/C/G/C/C/A/G, *p* = 0.022, OR = 1.294, 95% CI = 1.038–1.615; G/G/C/G/C/T/G/G, *p* = 0.0004, OR = 2.723, 95% CI = 1.535–4.829). There was no significant difference in the frequency distribution of the other haplotypes in the two groups.

## 4. Discussion

In recent years, the incidence of cardio-cerebrovascular disease has been increasing year by year. The CETP gene has been identified as an important independent risk factor of dyslipidemia and has become a hot research spot, and several mutations in this gene have been reported [17,18,19]. However, there is a big difference between the levels of serum lipids in different races, so taking into account the crucial role of CETP in lipid metabolism, we investigated the association of eight SNPs in this gene and the risk of dyslipidemia among the Uyghur and Kazakh Xinjiang national minorities.

Dyslipidemia is determined by a plurality of factors, and the study found that in the dyslipidemia group height, weight, BMI, WC, HC, WHR, SBP, DBP, TG, TC, LDL-C, FPG, ApoB, ApoB/ApoA were significantly increased compared with the control group, while HDL-C and ApoA were significantly reduced. In addition, the family history of dyslipidemia was higher, which indicated that genetic factors are important factors in determining dyslipidemia. Meanwhile, there was no significant difference between the smoking and drinking habits of the two groups. Obviously bad living habits are not the main cause of the abnormal blood lipids, and genetic factors are more important.

We found that in our study group eight SNPs located in the CETP gene (Table 2), were the most associated with dyslipidemia. Single factor logistic regression was found to have a significant correlation with the risk of dyslipidemia in five loci. They are rs711752 (*p* = 0.045, OR = 0.861, 95% CI = 0.745–0.996), rs12149545 (*p* = 0.0003, OR = 0.744, 95% CI = 0.633–0.876), rs708272 (OR = 0.867, 95% CI = 0.753–0.998), rs1800775 (OR = 1.161, 95% CI = 1.007–1.339), rs3764261 (OR = 0.754, 95% CI = 0.642–0.886). In this study, of the CETP gene eight SNPs we confirmed that at five genetic loci were associated with lipid parameters or dyslipidemia in a Uyghur and Kazakh national minority population in Xinjiang. The rs12149545 A allele and the rs711752 A allele play an active role in the development of dyslipidemia among the Xinjiang Uyghur and Kazakh ethnic groups. However, Kooner *et al*. [20] have found that the rs711752 minor allele of HDL-C was a risk factor (*p* < 0.05, OR = 1.73). The rs708272 T allele and rs3764261 T allele play an active role in the development and progression of dyslipidemia. This is consistent with Wang *et al*. [21] who reported on the Han population in Xinjiang, Radovica *et al*. [22] who reported for the US population and Goto *et al.* [23] reported Japanese data, but unlike Zhou *et al.* [24], who reported that for the Hainan Han group “CETP gene minor alleles are not genetic risk factors”. The C allele of rs1800775 is a genetic risk factor for dyslipidemia, which Radovica *et al*. [22] reported for the US population based on high TC results. Heid *et al*. [25] showed that HDL-C was a risk factor (*p* < 0.05, OR = 1.57), rs289714, but our study didn't find a correlation between them. We speculate therefore that the differences likely arise from the following two reasons: on the one hand, Kazakhs are a nomadic race that resides in mountainous areas where outdoor activities are impractical during the cold winter season. The main foods the Uyghur and Kazakh minorities consume are beef, wheat, dairy products and mutton, which contain high fat. The differences may be related to diet and lack of exercise. In another aspect, molecular studies have demonstrated that the Uyghur and Kazakh populations reside at the borders with countries where Caucasian and Asian peoples are mixed [26]. For example, Uyghurs have a mixture of 40% East Asian ancestry and 60% European ancestry [27], which may explain the similarities and differences between the results of this study and other studies. However the specific mechanism is still to be further studied.

Low HDL-C is another cause of dyslipidemia. Low HDL-C in primary prevention patients in the general population is an independent factor for coronary artery disease [28]. However, HDL metabolism is complex, and HDL functionality (e.g., cholesterol efflux from macrophages) is more important than HDL-C levels. That explains why in secondary prevention patients the prognostic factor is HDL functionality (cholesterol efflux) but not low HDL-C levels [29]. In summary, HDL quality (functionality) seems to be more important than HDL quantity (HDL-C concentrations) [30]. However, we have not examined in our study the effect of CETP polymorphisms on HDL functionality, only on TG, TC, LDL-C, HDL-C levels. This study also found that in the normal control group, 708272 CT genotype carriers’ TG levels are low CC genotype, TT genotype carriers LDL-C levels were lower than in subjects of the CC genotype, TC and HDL-C levels were higher than in the CT genotype. This is consistent the the Ellman [31] research on black South Africans, but unlike the results of Zhou *et al*. [32] for the Hei Yi Zhuang population in Guangxi; rs711752 AG genotype carriers’ TG levels were lower than those of the GG genotype, AA genotype carriers LDL-C level was lower than those of the GG genotype, AA genotype carriers’ HDL-C levels are relatively high in the AG genotype, consistent with Radovica *et al*. [22] results. Dyslipidemia group 1800775 AC genotype carriers of high TG levels are AA genotype, different from the data of Lu *et al*. [33] study of the multi-ethnic population of Singapore; rs3764261 TT genotype carriers with high TC levels are GG genotype carriers, inconsistent with Chang *et al*. [34] studies. rs711752 AA genotype carriers of TC, LDL-C levels are relatively high AG genotype; rs12149545 AA genotype carriers of TC, LDL-C levels is high compared with GG genotype, different from the results of Suet Nee [35]. These results indicate a close correlation between CETP gene polymorphism and serum lipid levels.

CETP belongs to the lipid transfer and lipopolysaccharide gene family. It is a hydrophobic glycoprotein that mediates the exchange of cholesterol ester and TG in HDL with LDL-C or very low density lipoprotein, which regulates the concentration, composition and particle size of HDL. It is reported in the literature that CETP gene polymorphism can lead to the mutation of the CETP gene, thereby causing CETP deficiency, and CETP in HDL is important to the processing of small particles. In the condition of high CETP, the efficiency of HDL transfer TG is increased, which leads to the reduction of TG, and the HDL particles will be cleared rapidly, and the HDL-C level is reduced [36]. From the results, we speculate that rs711752, rs708272, and rs12149545 three locus polymorphism may mainly act by adjusting the TC, LDL-C level can affect The Xinjiang Uygur and Kazak people’s dyslipidemia, while rs3764261 locus polymorphism may be mainly affected by the TC level. Meanwhile it suggests the CETP gene polymorphism and lipid component differences may be due to genetic factors or regional differences.

Linkage disequilibrium (LD), also known as allelic association, refers to the non-random association between two alleles on the same chromosome. When the same chromosome, the probability of the existence of the same gene at the same time than the random distribution of the population, while the probability that these locis are in the LD state. When D′ = 1 when completely LD, described did not occur between the loci of recombination, when D′ = 0 means no LD or linkage equilibrium; when 0 < D′ < 1, indicating the occurrence of recombination or mutation among loci, *r*^2^ and D′ the same meaning. When *r*^2^ > 0.33, suggesting strong LD. CETP gene rs12149545, rs3764261, rs1800775, rs711752, rs708272 five loci in linkage disequilibrium. These sentences do not mean anything-rewrite at the same time, the results showed that two rs12149545 and rs3764261, rs708272 and rs711752 groups exist between strong LD. The association of CETP gene was also demonstrated in other studies [37,38], Hinds *et al.* [37] studies showed that the haplotype of CETP four loci was significantly correlated with the level of HDL-C.

Numerous recent studies have showed that SNP linkage disequilibrium between the pair is an unstable number [39,40]. The linkage disequilibrium depends in a complex way on recombination, geography, mutation, and the demographic history of the population under study. The human genome seems to be organized like a string of pearls, with a non-recombinant recombination hotspots and intervention block [41,42]. If this model is true then additional SNP measurements should not create too many separate new haplotypes, and should be adjusted to the existing spectrum. Linkage disequilibrium pairwise SNP depends on the allelic composition and frequency of the few haplotypes and may accordingly vary over a wide range. Functional diversity caused by different alleles may in such a situation reveal itself only by association with distinct haplotypes. Instead, association with SNPs may refer to a mixture of quite different chromosomal haplotypes that happen to the same carry SNP alleles. This text looks like it was copied exactly from some other source.

In the Xinjiang Uyghur and Kazakh ethnic groups, using SHEsis software to construct and analyze the loci haplotypes the data, the results showed that the two groups of the eight loci of the CETP gene were more than 0.01 of the 17 species, five of which were statistically significant (*p* < 0.05); It’s suggested that A/T/A/A/T/C/A/G, A/T/A/A/T/T/G/A and G/G/A/G/C/C/G/G are the protective factors in the development of dyslipidemia the Xinjiang Uyghur and Kazakh minority population. It may reduce the risk of abnormal dyslipidemia in the two ethnic groups. Nevertheless, the G/G/C/G/C/C/A/G and G/G/C/G/C/T/G/G risk factors that the two nationalities have increased risk of suffering from dyslipidemia is 1.294, 2.723 times, respectively. Like to the results of Meiner [43], rs12149545, rs708272, rs1800775 and other genotypes will increase the haploid constructing risk of the people suffering from myocardial infarction is about 4-6 times Makes no sense. In summary, we demonstrated a small number of CETP gene SNP haplotypes in a random sample of the Uyghurs and Kazakhs population. Our study found a clear association of several loci with functional dyslipidemia, but they can explain only a small part of the genetic variation segregation in the population. At the same time, the effect of haplotype combinations on the phenotype has multiple aspects. On the one hand, it may be a synergistic or antagonistic effect of genes or an allele of a gene, on the other hand is a certain alleles play a leading role. Because of the complex interactions between genes, these results need to be further analyzed and verified at a later stage.

## 5. Study Limitations

There are >180 SNPs for the CETP gene in dbSNP, therefore a large number of SNPs are not covered in this study. Our study is also on a single gene, CETP, so further evaluation of the tSNPs among multiple genes and loci set of cases and controls is needed in the future in order to better clarify dyslipidemia associations with gene polymorphism.

In both the Kashi and Yili Prefectures, most Uyghurs and Kazakhs reside in low-income rural communities where public health resources are very limited. For instance, in the Uyghur concentrated Jiashi county, more than 92% of Uyghurs live on 1.00 US dollar a day or less, in stark contrast to life in the US $1.00 dollars a day, it is the national average of 15.9% in 2005 [44,45]. Although our study didn’t incorporate all of the CETP gene locus this study suggests that several loci in the Uyghur and Kazak populations are clearly related to dyslipidemia. This is the first report of an association between the multiple CETP loci and dyslipidemia in two major minorities in Northwest China, which may provide a new way of thinking about the diagnosis, prevention and treatment of dyslipidemia at the genetic level in the Uyghur and Kazakh national minority populations. However, the relationship between CETP gene polymorphism and dyslipidemia is very complex, and CETP gene is influenced by environmental factors and metabolic factors in the process of lipid abnormality. At the same time, due to the differences of race and region, the method of research design and other factors that affect the different conclusions of the research, the sample needs to be expanded and the interaction between genes and the gene environment and the interaction of multiple genetic susceptibility genes in different reaction systems studied,, and this will be our research topic in the future, in order to provide a better scientific basis for the prevention and treatment of clinical diseases through relevant research.

## 6. Conclusions

The polymorphism of CETP gene rs708272, rs3764261, rs1800775, rs711752, rs12149545 was closely related to the occurrence of dyslipidemia among the Xinjiang Uyghur and Kazakh ethnic groups; The frequencies of the rs708272 T alleles, rs3764261 T alleles rs711752 A alleles and rs12149545 A alleles are protective factors against dyslipidemia in the Uyghur and Kazakh population. However, the rs1800775 C allele showed risk factors. Our results provide a new way of thinking about the diagnosis, prevention and treatment of dyslipidemia at the genetic level in the Uyghur and Kazakh national minority populations, while providing a genetic basis for the prevention and treatment of cardiovascular and cerebrovascular diseases.
